# Allergy manifestations to fish in younger children

**DOI:** 10.1186/2045-7022-5-S3-P163

**Published:** 2015-03-30

**Authors:** Adnan Bajraktarevic, Faruk Alendar, Elvedin Landzo, Armina Rovcanin, Emina Beslagic, Lejla Kumasin, Elvira Lokmic, Amira Skopljak, Zijo Begic, Amina Selimovic, Lutvo Sporisevic, Branka Djukic, Alisa Abduzaimovic

**Affiliations:** 1 Pediatrics Department, Public Health Institution of Health Center Sarajevo, Sarajevo, Bosnia and Herzegovina; 2 Dermatologic Clinic, Sarajevo, Bosnia and Herzegovina; 3 Clinical Medical Center Sarajevo, Sarajevo, Bosnia and Herzegovina; 4 Pharmaceutical Faculty Sarajevo Department for Pharmacology, Sarajevo, Bosnia and Herzegovina; 5 Pediatrics Clinic Sarajevo, Sarajevo, Bosnia and Herzegovina; 6 Pediatrics Department, First Medical Aid New Sarajevo, Sarajevo, Bosnia and Herzegovina; 7 Biochemical Allerology Laboratory Tesanj, Tesanj, Bosnia and Herzegovina

## Introduction

Allergy to fish is relatively common in children. In addition to skin manifestations are present and the sensitivity of the intestine, with diarrhea and present the general weakness of the child.

## Aims

The aim of this study was to evaluate this allergic manifestation in the youngest population, regardless of whether they are allergy to fish originated from the sea or river or lake.

## Methods

Data were collected to determine the preschool children patients' allergies documented in the medical record. Four hundred and fifty two children with at least one documented food allergy and thirty seven (8.19%) children who had a fish allergy while hospitalized or during exanimations and curing in pediatrics primary practice for seven years period from 2007 to 2014 in Sarajevo.

## Results

Dermatologic signs and symptoms were the most common in 31 cases (83.78%). Urticaria were seen in less than a fifth of 7 cases in preschool children of all signs on skin. Also seen were erythema 18.91 %, face or lip swelling 10.81%, extremity edema 8.11%, pruritis 67.57%, and other rashes 45,94% . Second were gastrointestinal (nausea, vomiting, and abdominal pain), third neurologic (aura, irritability, lethargy, disorientation, dizziness, tremor, syncope, and seizure), and fourth cardiovascular (hypotension, hypoperfusion, tachycardia, bradycardia, and asystole) initial manifestations were much less common in 5.41% in children. Respiratory abnormalities were the last were most often observed initial signs and symptoms in 2.70%.

## Conclusions

Allergic reactions to fish in children is a very serious issue which has to be approached systematically and promptly for treatment. No significant differences in the clinical presentation and treatment between freshwater fish and marine fish. In Bosnia and Herzegovina are not described fatal reactions in children from an allergy to fish in this seven-year period.

